# Hygrothermal Stress Analysis of Epoxy Molding Compound in Fan-Out Panel-Level Package Based on Experimental Characterization and Structural Sensitivity

**DOI:** 10.3390/polym17152034

**Published:** 2025-07-25

**Authors:** Yu-Chi Sung, Chih-Ping Hu, Sheng-Jye Hwang, Ming-Hsien Shih, Wen-Hsiang Liao, Yong-Jie Zeng, Cheng-Tse Tsai

**Affiliations:** 1 Department of Mechanical Engineering, National Cheng Kung University, Tainan 70101, Taiwan; 2 Department of Mechanical Engineering, Southern Taiwan University of Science and Technology, Tainan 710301, Taiwan; 3 Packaging Product Simulation and Design Division, Innolux Corporation, Tainan 744092, Taiwan; 4 Testing Center, Innolux Corporation, Tainan 744092, Taiwan

**Keywords:** fan-out panel-level packaging, moisture effect, hygrothermal stress, structural sensitivity analysis, reliability

## Abstract

As semiconductor devices demand higher input–output density and faster signal transmission, fan-out panel-level packaging has emerged as a promising solution for next-generation electronic systems. However, the hygroscopic nature of epoxy molding compounds raises critical reliability concerns under high-temperature and high-humidity conditions. This study investigates the hygrothermal stress of a single fan-out panel-level package unit through experimental characterization and numerical simulation. Thermal–mechanical analysis was conducted at 100 °C and 260 °C to evaluate the strain behavior of two commercial epoxy molding compounds in granule form after moisture saturation. The coefficient of moisture expansion was calculated by correlating strain variation with moisture uptake obtained under 85 °C and 85% relative humidity, corresponding to moisture sensitivity level 1 conditions. These values were directly considered into a moisture -thermal coupled finite element analysis. The simulation results under reflow conditions demonstrate accurate principal stress and failure location predictions, with stress concentrations primarily observed at the die corners. The results confirm that thermal effects influence stress development more than moisture effects. Finally, a structural sensitivity analysis of the single-package configuration showed that optimizing the thickness of the dies and epoxy molding compound can reduce maximum principal stress by up to 12.4%, providing design insights for improving package-level reliability.

## 1. Introduction

With the rapid advancement of technology, the demand for 3C (computer, communication, and consumer electronics) products continues to grow steadily. To meet this demand, semiconductor devices are trending toward high-density integration and miniaturized packaging. Among emerging packaging technologies, fan-out panel-level packaging (FOPLP) presents substantial advantages, particularly in cost efficiency and process scalability. By enabling processing of larger panels, FOPLP significantly reduces the unused edge area typically observed in wafer-level packaging, thus enhancing material utilization and manufacturing throughput.

**Figure 1:**
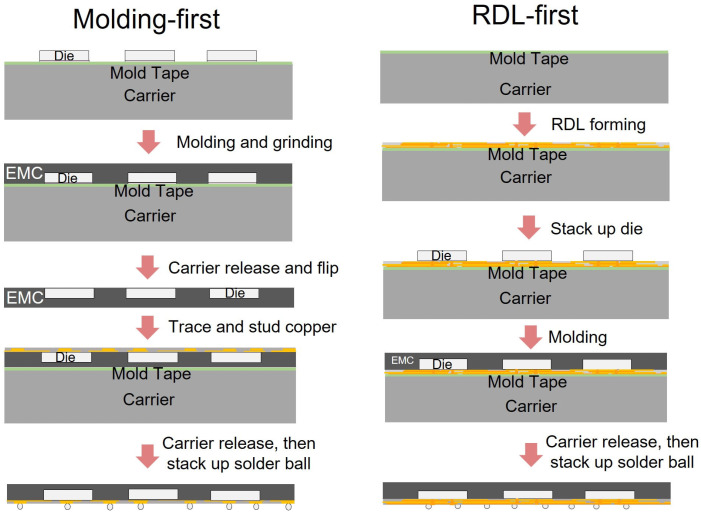
Process flowchart of molding-first and RDL-first fan-out process.

FOPLP can generally be categorized into two main process flows: the redistribution layer (RDL)-first process and the molding-first process [1,2]. In the RDL-first approach, the RDL and dies are first placed onto the carrier, followed by encapsulation and carrier removal. Conversely, the molding-first process begins by molding the dies onto the carrier, followed by removal of the carrier board, and then stacking a copper layer beneath the dies [3]. This study focuses on the molding-first approach in FOPLP, analyzing its process characteristics and implications. Figure 1 illustrates the process flowcharts for both methods.

In recent years, moisture-induced reliability concerns have become increasingly prominent in electronic packaging, primarily due to polymer materials’ hygroscopic and porous nature. During packaging, storage, and transportation, moisture can infiltrate the encapsulant and diffuse into internal structures. Once inside, it may cause differential expansion between materials, leading to stress accumulation and an increased risk of delamination, particularly during thermal processes such as reflow soldering [4]. Therefore, accurately modeling moisture diffusion is critical for predicting failure mechanisms and improving overall package reliability.

Moisture transport within polymer-based encapsulants is primarily governed by diffusion mechanisms, which can be described using the transient form of Fick’s Law. Wong [5] proposed a humidity-based modeling approach to evaluate delamination failure in electronic packages, using finite element analysis (FEA) to simulate moisture concentration across heterogeneous material interfaces. Chen [6] applied the Taguchi method to investigate moisture behavior in plastic ball grid array (PBGA) packages under varying temperature and humidity conditions, revealing that increased moisture uptake correlates with more severe warpage. Considering the effect of vapor pressure generated by high-temperature water vapor, Tee [7] developed a coupled model that incorporates humidity, temperature, and vapor pressure effects. The results showed that moisture and vapor pressure contribute comparably to stress, while temperature-induced thermal strain remains the dominant factor. To monitor real-time stress evolution during reflow, Lwo [8] employed piezoresistive sensors in BGA packages and observed a sharp stress drop during the onset of delamination.

Tsai [9] developed a comprehensive moisture diffusion model for flip chip ball grid array (FCBGA) packages using ANSYS simulations. The study revealed localized moisture retention at material interfaces, which may compromise adhesion and trigger failure. Wu [10] evaluated the reliability of fan-out wafer-level packaging (FOWLP) using FEA and demonstrated that peak stress occurs at the corner interface between the epoxy molding compound (EMC) and silicon. In a follow-up study, Chiu [11] conducted double cantilever beam (DCB) tests on EMC and polyimide (PI) films, confirming that moisture absorption significantly weakens interfacial adhesion. In parallel, Chen [12] investigated the effect of printed circuit board (PCB) thickness in system-in-package (SiP) assemblies and found that thinner PCBs intensify moisture concentration and internal stress due to shortened diffusion paths.

Recent studies have also addressed various aspects of FOPLP reliability. Jiang [13] focused on the reliability of power devices under pressure cooker testing, emphasizing delamination behavior due to moisture uptake. Liang [14] conducted comprehensive analyses of warpage and residual stress in panel-level packages, considering the effects of process steps such as molding and debonding. However, these studies do not directly measure the coefficient of moisture expansion nor simulate the coupled interaction between moisture diffusion and thermal–mechanical behavior during reflow.

Compared with these prior studies, the present work addresses several key limitations in current FOPLP reliability research. Most existing analyses consider moisture diffusion and mechanical stress separate phenomena, often without integrating experimentally validated material properties into the simulation. In addition, few studies integrate structural sensitivity analysis to evaluate how variations in package geometry affect local stress concentrations under actual conditions such as moisture sensitivity level testing and reflow soldering. This research indicates a lack of comprehensive methodologies that capture the coupled effects of moisture and temperature while reflecting actual material behavior.

To overcome these limitations, this study proposes a simulation framework that combines experimentally measured coefficients of moisture expansion with a coupled moisture and thermal finite element model. This enables accurate prediction of principal stress distribution and failure-prone locations under JEDEC standard J-STD-020 MSL 1 [15]. Furthermore, a parametric sensitivity analysis examines the influence of overmold, dies, and WMF thickness on local stress. The proposed approach offers a novel and practical methodology for improving FOPLP reliability by bridging experiment characterization with physics-based simulation and design evaluation.

## 2. Theory

### Moisture Diffusion Model

It is assumed that diffusion flux of moisture does not change with time. Since the transfer of moisture is similar to heat transfer, it is described using Fick’s first law.

(1) $$\frac{\partial C_{\mathrm{ab}}}{\partial t}=D(\frac{\partial^{2}C_{\mathrm{ab}}}{\partial x^{2}}+\frac{\partial^{2}C_{\mathrm{ab}}}{\partial y^{2}}+\frac{\partial^{2}C_{\mathrm{ab}}}{\partial z^{2}})$$

$C_{\mathrm{ab}}$: absolute concentration; *x*, *y*, *z*: spatial coordinates; *D*: moisture diffusivity; *t*: time.

Therefore, the concept of relative humidity (*W*) is applied to the absolute humidity equation due to concentration discontinuity at the interface between two materials.

(2) $$\frac{\partial W}{\partial t}=D(\frac{\partial^{2}W}{\partial x^{2}}+\frac{\partial^{2}W}{\partial y^{2}}+\frac{\partial^{2}W}{\partial z^{2}}),\;W\;=\frac{C_{\mathrm{ab}}}{C_{\mathrm{sat}}}$$

W: relative concentration; *C_sat_*: saturated moisture concentration.

In addition, a constant temperature and humidity chamber can be used to conduct moisture experiments to measure material parameters about humidity, such as moisture diffusivity (*D*), saturated moisture concentration (*C_sat_*), and the coefficient of moisture expansion (CME). Based on Fick’s law, saturated moisture concentration and moisture diffusivity can be expressed as

(3) $$C_{\mathrm{sat}}=\frac{M_{\mathrm{sat}}}{l\cdot w\cdot h\;(100-\mathrm{vol}\%\;\mathrm{of}\;\mathrm{moisture})}$$

(4) $$\frac{M_{t}}{M_{\mathrm{sat}}}=1-\sum_{n=0}^{\infty }\frac{8}{{\left(2n+1\right)}^{2}\pi^{2}}\exp(\frac{-D{\left(2n+1\right)}^{2}\pi^{2}t}{{L_{\mathrm{Thickness}}}^{2}})$$

*M_sat_*: the saturated mass during absorption; *l*, *w*, *h*: the length, width and height dimensions of the sample; vol% of moisture: the percentage of moisture in the sample; $M_{t}$: the mass at time *t*, *D*: moisture diffusivity; *t*: time; *L_Thickness_*: sample thickness.

The coefficient of moisture expansion is obtained by linear regression on the changes in dimensions and weight measured by thermogravimetric analysis (TGA) and thermal–mechanical analysis (TMA).

In addition, moisture–thermal strain equation can be expressed as

(5) $$\{\varepsilon_{\mathrm{Tot}}\}=\{\varepsilon_{\mathrm{el}}\}+\{\varepsilon_{\mathrm{th}}\}+\{\varepsilon_{h}\}={\left[S\right]}^{-1}\{\sigma \}+\alpha_{T}\;\Delta T+C_{\mathrm{sat}}\;ꞵ\Delta W$$

(6) $${\left[S\right]}^{-1}=\left[\begin{array}{cccccc} 1/E_{x} & -v_{\mathrm{xy}}/E_{x} & -v_{\mathrm{xz}}/E_{x} & 0 & 0 & 0\\ -v_{\mathrm{yx}}/E_{y} & 1/E_{y} & -v_{\mathrm{xz}}/E_{y} & 0 & 0 & 0\\ -v_{\mathrm{zx}}/E_{z} & -v_{\mathrm{zy}}/E_{z} & 1/E_{z} & 0 & 0 & 0\\ 0 & 0 & 0 & 1/G_{\mathrm{xy}} & 0 & 0\\ 0 & 0 & 0 & 0 & 1/G_{\mathrm{yz}} & 0\\ 0 & 0 & 0 & 0 & 0 & 1/G_{\mathrm{xz}} \end{array}\right]$$

{*ε_Tot_*}: moisture–thermal coupled strain; {*ε_el_*}: elastic strain; {*ε_th_*}: thermal strain; {*ε_h_*}: moisture strain; [S]: stiffness matrix; {*σ*}: moisture-thermal coupled stress; *α_T_*: thermal expansion coefficient; Δ*T*: temperature difference; *C_sat_*: saturated moisture concentration; *ꞵ*: coefficient of moisture expansion; Δ*W*: humidity difference; $E_{x}$, $E_{y}$, and $E_{z}$: young’s modulus along x, y, and z directions, separately; $v_{\mathrm{xy}},\;v_{\mathrm{zx}},\;v_{\mathrm{yz}}$: Poisson ratios of material; $G_{\mathrm{xy}},\;G_{\mathrm{zx}},\;G_{\mathrm{yz}}$: shear modulus.

## 3. Moisture Experiment

In this section, EMC is used to conduct moisture experiments. The thermal–mechanical analysis (TMA) results of these moisture experiments are discussed. Additionally, the coefficient of moisture expansion will be calculated from the experiment results to be used in subsequent moisture simulations.

### 3.1. Procedures of Moisture Experiment

**Figure 2:**
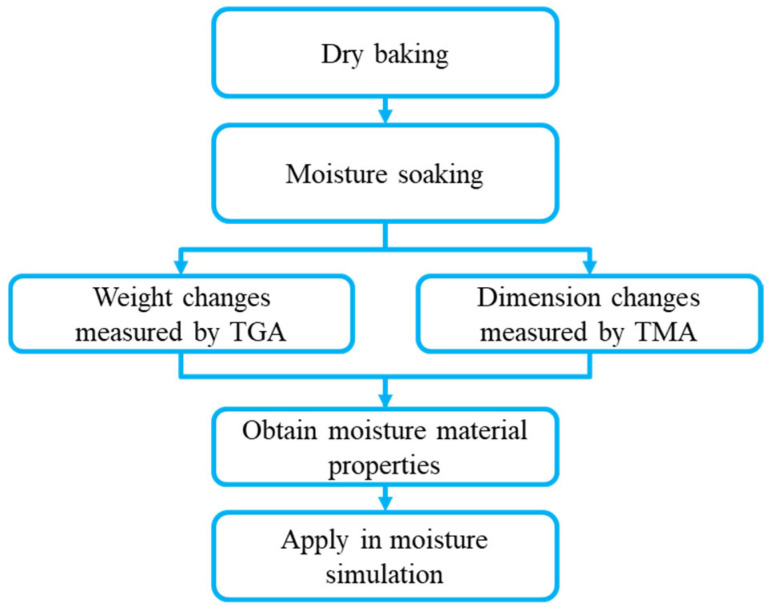
Schematic of moisture experiment procedures.

The experiment procedure for evaluating the moisture behavior of EMC materials followed the methodology proposed by Hsu [16], as illustrated in Figure 2. Initially, the fully cured EMC, shaped into thin sheets, was baked at high temperature to eliminate pre-existing moisture absorbed from the ambient environment.

The dried samples were then placed in a temperature and humidity chamber at 85 °C and 85% relative humidity (RH) for up to 168 h to allow moisture absorption.

After saturation, the samples were subjected to both thermogravimetric analysis (TGA) and thermomechanical analysis (TMA). TGA was used to quantify the weight loss resulting from moisture desorption during isothermal heating, while TMA was employed to measure dimensional changes caused by moisture loss under the same thermal conditions. By integrating the results from both analyses, the coefficient of moisture expansion (CME) was calculated.

**Figure 3:**
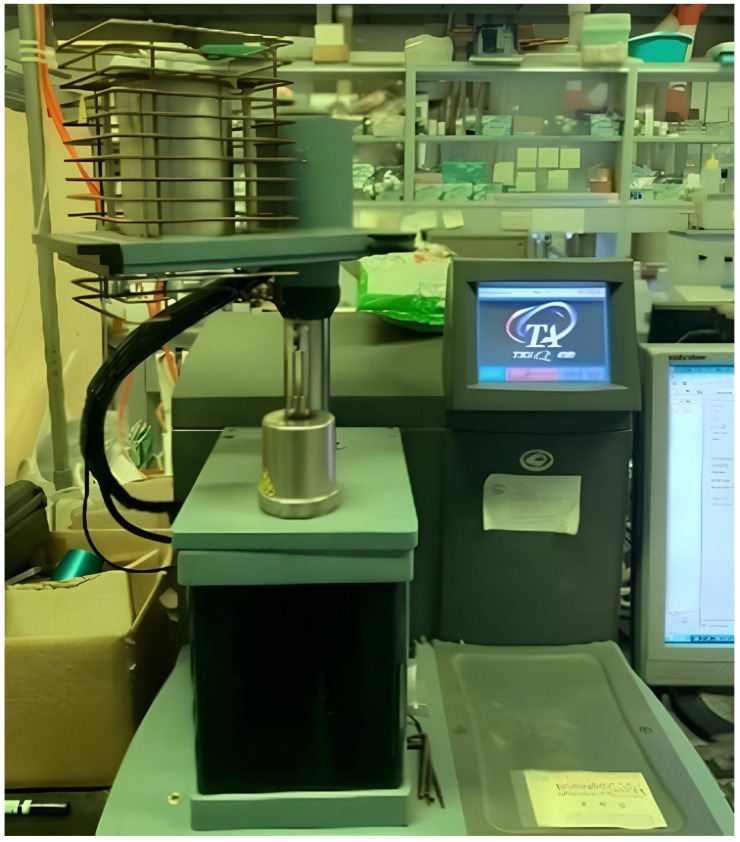
Schematic of thermal–mechanical analyzer (Q400 TMA).

**Figure 4:**
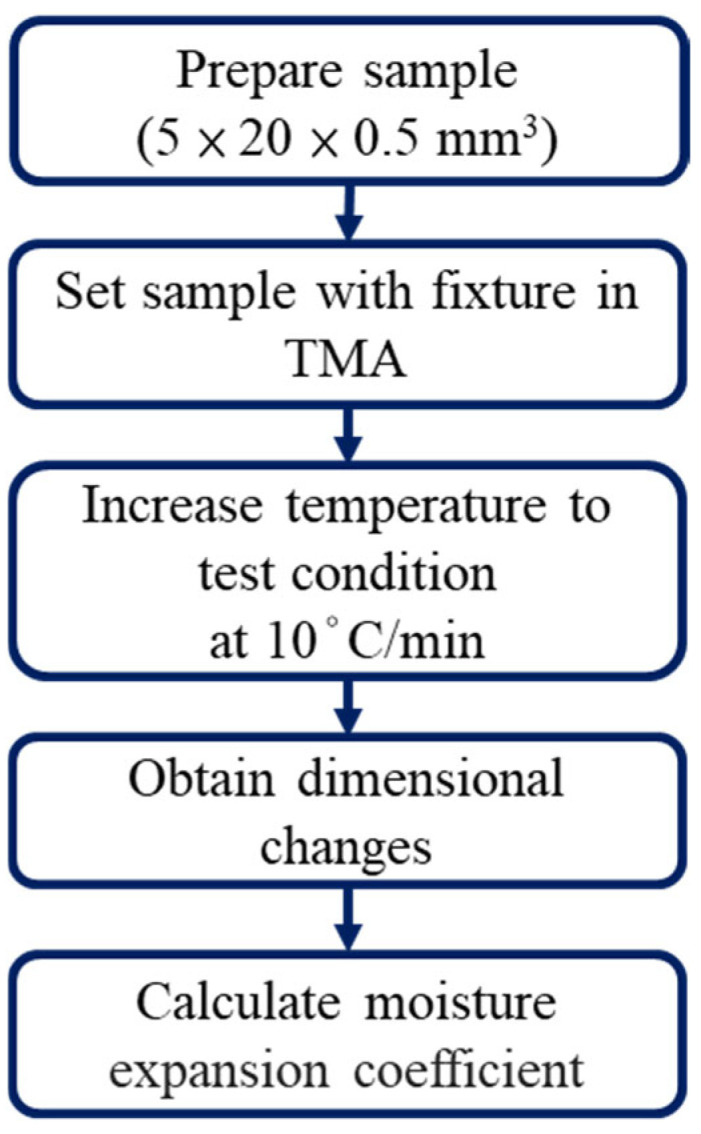
Schematic of TMA experiment procedures.

**Figure 5:**
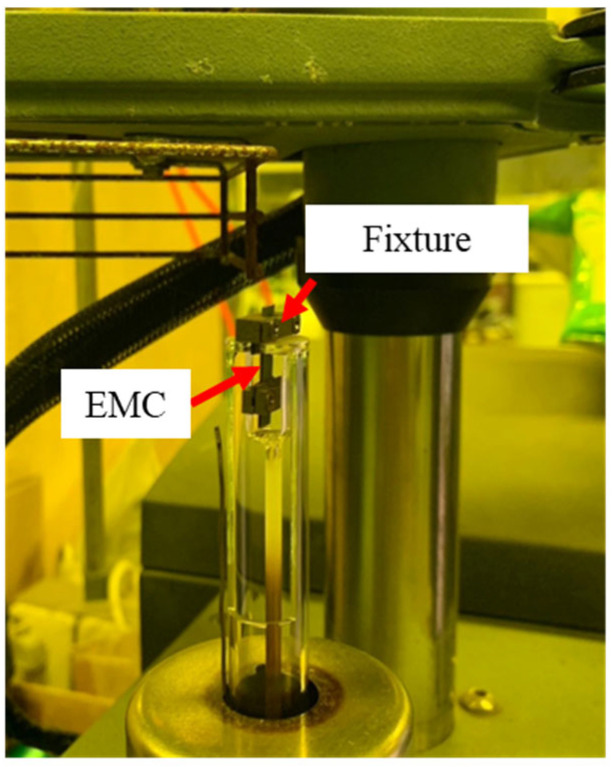
Location of EMC and fixture in TMA.

The moisture absorption and TGA experiments were primarily conducted by Innolux Corporation, Tainan, Taiwan. Therefore, this study focuses on the TMA procedures and results. The TMA equipment (Q400, TA Instruments, New Castle, DE, USA) and experimental process are shown in Figure 3 and Figure 4. As illustrated in Figure 5, samples with dimensions of 5 × 20 × 0.5 mm^3^ were clamped onto the TMA stage. The samples were heated at a rate of 10 °C/min to the target temperature and kept for 300 min to observe the dimensional changes associated with moisture desorption.

**Figure 6:**
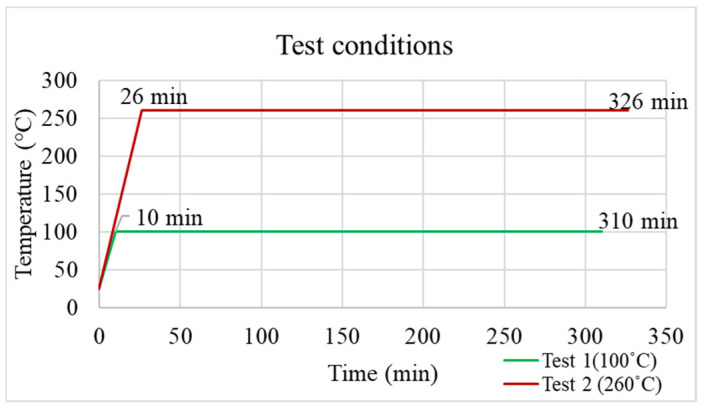
Different testing conditions for TMA experiments.

The applied temperature profiles for the TMA experiments are shown in Figure 6. Two testing temperatures, 100 °C and 260 °C, were used, both with the same heating rate and holding duration. The grey line indicates the temperature ramping process reaching 100 °C within 10 min. These conditions were designed to investigate strain behavior under different thermal loads. In addition, two EMC materials (EMC 1 and EMC 2) were examined to compare their hygroscopic and thermomechanical responses.

### 3.2. TMA Experiment Results

**Figure 7:**
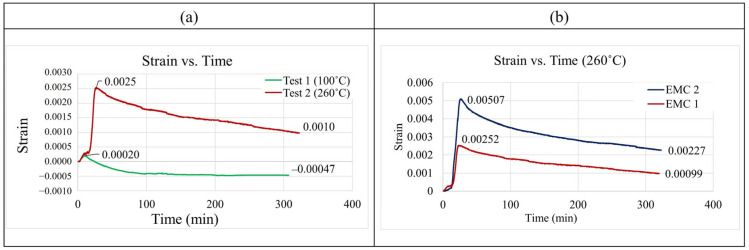
(**a**) The strain variation at different testing conditions. (**b**) The strain variation with the two EMC materials.

Figure 7a shows the strain variation under different testing conditions. An initial increase in strain is observed as the temperature rises, primarily due to thermal expansion of the EMC and surrounding materials. Once the structure reaches the elevated temperature, the strain gradually decreases as the internal moisture content in the EMC is reduced through temperature-driven diffusion and desorption. Figure 7b illustrates the strain variation for two types of EMC material (EMC 1 and EMC 2). Compared to EMC 1, EMC 2 exhibits more significant strain variation, indicating a stronger response to the combined effects of temperature and moisture.

### 3.3. Coefficient of Moisture Expansion (CME) Calculations

**Figure 8:**
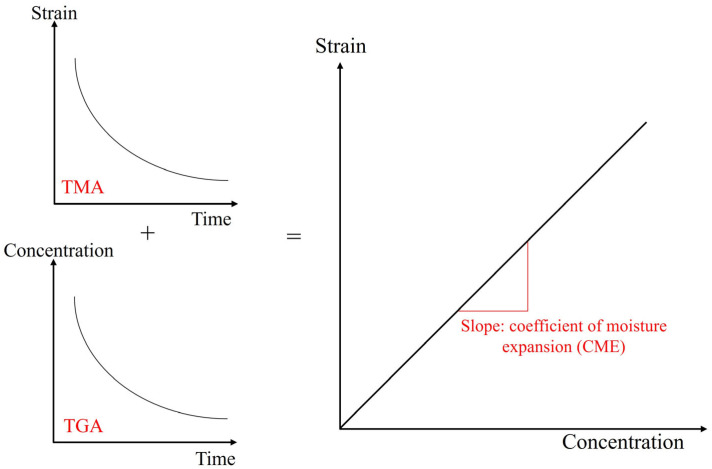
Calculation procedure for coefficient of moisture expansion.

The coefficient of moisture expansion was calculated using the method proposed by Wong [17]. As shown in Figure 8, the obtained slope represents the coefficient of moisture expansion.

**Figure 9:**
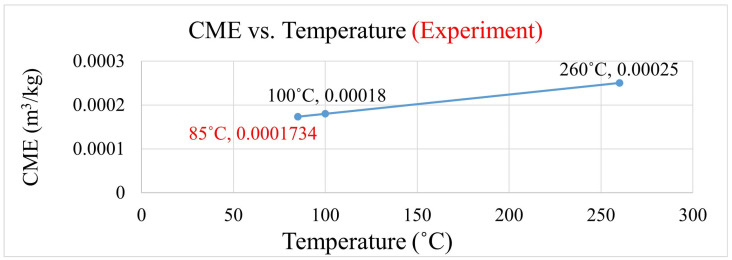
Coefficient of moisture expansion at different temperatures.

Coefficient of moisture expansions for EMC 1 material at the two testing conditions (100 °C and 260 °C) were calculated to be 1.8 × 10^−4^ and 2.5 × 10^−4^ (m^3^/kg), respectively. Since the moisture sensitivity levels (MSLs) were calculated at 85 °C, the coefficient of moisture expansion was obtained through extrapolation and was found to be 1.7 × 10^−4^ (m^3^/kg), as shown in Figure 9.

Additionally, the coefficient of moisture expansion (CME) for EMC 2 at high temperature (260 °C) is measured to be 4.3 × 10^−4^ (m^3^/kg). Compared to EMC 1, EMC 2 exhibits a higher CME value, indicating that different EMC materials respond differently to moisture-induced expansion. However, since the experimental data for EMC 1 is more complete and EMC 1 is the material actually used in the packaging process, all subsequent hygrothermal simulation analyses will incorporate the experimentally measured properties of EMC 1.

## 4. Hygrothermal Stress Analysis

The coefficient of moisture expansion for EMC materials, calculated from the moisture experiments, was used for simulation analysis. A geometric model, mesh size, analysis settings, and material properties are introduced in this section. We observed the moisture diffusion and moisture–thermal stress distribution during moisture sensitivity level tests (MSL tests).

**Figure 10:**
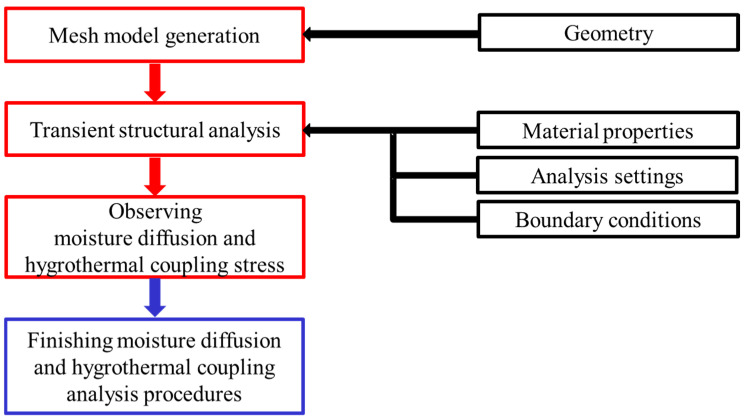
Analysis procedures of hygrothermal stress analysis.

The analysis procedure is illustrated in Figure 10.

### 4.1. Geometry and Mesh Generation

**Figure 11:**
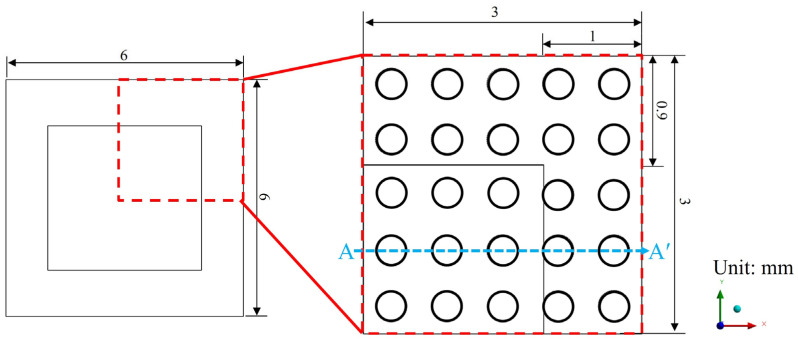
Top view of single-package model.

**Figure 12:**
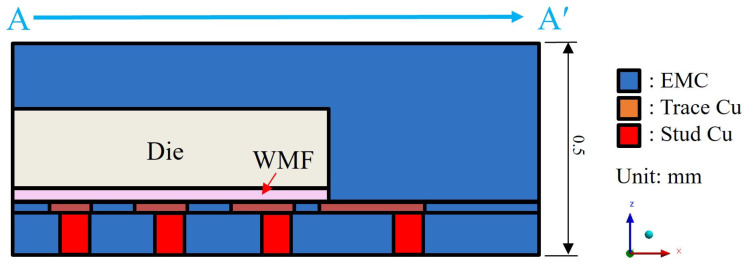
Schematic of sectional view of quarter single-package model.

As shown in Figure 11 and Figure 12, a quarter single-package model was established to simulate the representative structure of a typical FOPLP. Figure 11 illustrates the top view of the model, with the dashed line A–A′ indicating the cross-sectional location depicted in Figure 12. The model consists of an EMC, silicon die, WMF, copper trace layer, and copper stud layer. In particular, the copper trace layer includes both copper wiring and the surrounding EMC. Due to the complex geometry and fine features of the copper wiring, direct modeling is computationally expensive. To reduce meshing complexity and simulation time, the rule of mixtures was adopted to calculate the equivalent material properties of the trace layer based on the copper volume fraction. In this study, the copper coverage of the equivalent trace layer was set to 59%.

### 4.2. Analysis Settings

The temperature and humidity environment used in this study followed moisture sensitivity level 1 (MSL 1), with conditions set at 85 °C/85% RH for 168 h. This level represents the most stringent conditions in MSL 1.

**Figure 13:**
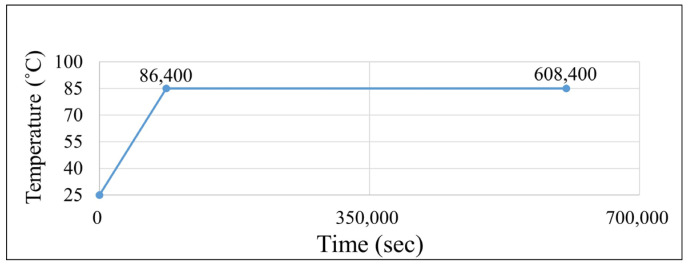
Temperature profile setting of MSL 1 testing conditions.

Figure 13 shows the temperature profile applied to a single package during MSL testing. The temperature was first ramped from room temperature (25 °C) to 85 °C, followed by a constant-temperature dwell at 85 °C.

Regarding the humidity boundary conditions, two main settings were applied. First, the initial moisture concentration within the entire package was set to zero, simulating a dry state after the baking process. Second, the surfaces of the package exposed to the external environment were set to 85% RH to mimic real-world conditions.

**Figure 14:**
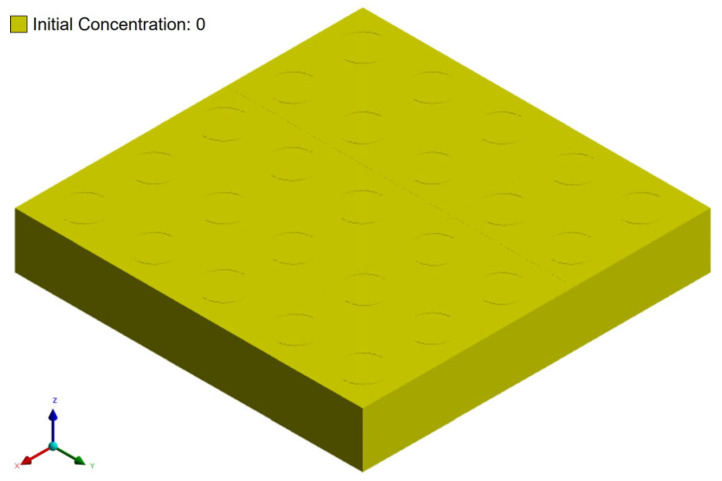
Initial concentration setting of package model.

**Figure 15:**
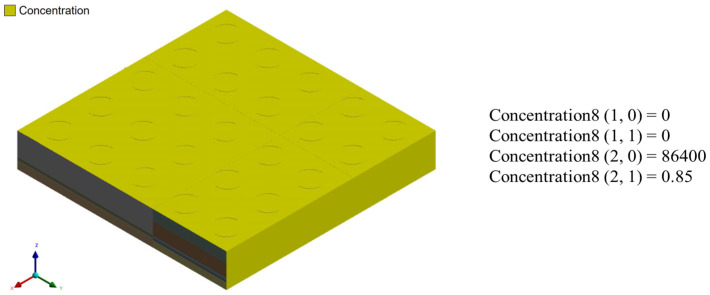
Concentration setting during moisture diffusion process.

To more accurately replicate the gradual moisture absorption that occurs at the surface in a humid environment, the boundary condition was defined such that the moisture concentration on the exposed surfaces increased from 0% RH to 85% RH over a 24-h period. The detailed humidity boundary conditions are illustrated in Figure 14 and Figure 15.

### 4.3. Material Properties

The material properties were divided into temperature- and humidity-dependent parameters. The Innolux Corporation provided the thermal–mechanical properties, and the humidity-related parameters were based on the moisture experiments and references to Hong [18] and Gao [19], as shown in Table 1.

### 4.4. Mesh Convergence Analysis

**Figure 16:**
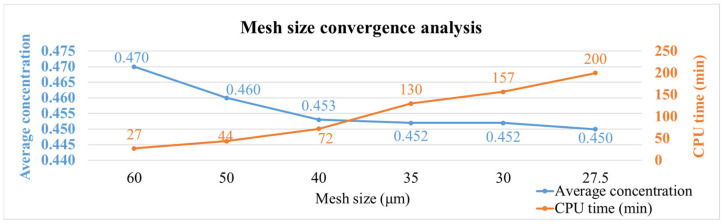
Moisture concentration results with different mesh sizes.

Different mesh models were established for the single-package model to observe the moisture concentration and maximum principal stress results. Table 2 shows the number of elements for each mesh configuration, and Figure 16 presents the moisture concentration results corresponding to different mesh sizes. In Figure 16, the blue line represents the average moisture concentration of the entire package, while the orange line indicates the total simulation time. It can be observed that when the mesh size is between 35 μm and 27.5 μm, the moisture diffusion results begin to stabilize, with minimal variation in average concentration despite finer meshing. Therefore, a mesh size of 35 μm was selected for subsequent simulations as a balance between accuracy and computational efficiency.

**Figure 17:**
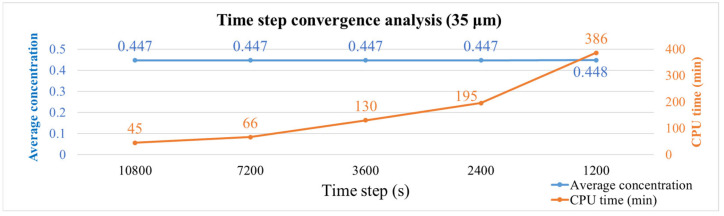
Moisture concentration results with different time steps.

In addition to mesh refinement, a time step convergence study was also conducted since the simulation involves transient structural analysis. As shown in Figure 17, overly large time steps may lead to significant numerical errors at each step, whereas excessively small time steps substantially increase computation time. The plot in Figure 17 compares the evolution of average moisture concentration under different time step sizes. The results indicate that the average concentration remains consistent across various settings, while larger time steps offer a clear advantage in reducing simulation time. Based on these results, a time step of 10,800 s was selected in combination with the 35 μm mesh for all subsequent coupled moisture–thermal analyses.

**Figure 18:**
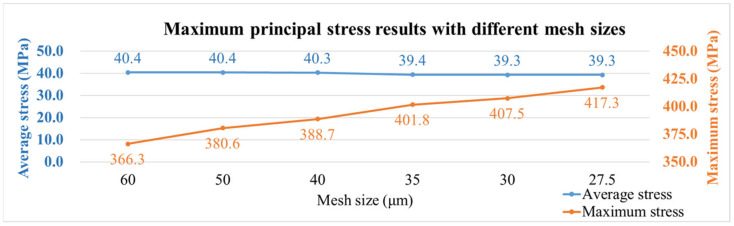
Maximum principal stress results with different mesh sizes.

**Figure 19:**
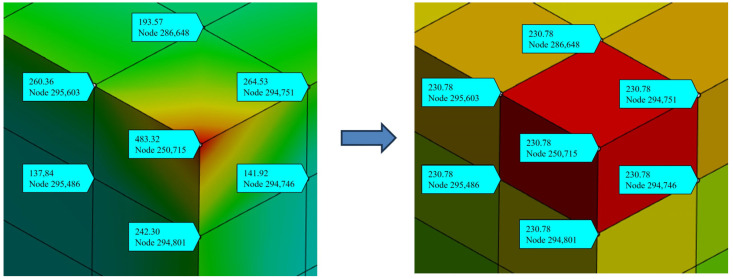
Interpretation method of maximum principal stress results.

Additionally, maximum principal stress was used to observe convergence results. Figure 18 shows the maximum principal stress for different mesh sizes, with the two curves representing the average stress of the package (blue) and the maximum stress value (orange). It was observed that as the mesh size decreases, the average stress value approaches stability, while the maximum stress value increases with smaller mesh sizes. The maximum stress values are concentrated at the corners of the die. To reduce the effects of stress concentration, the average value of all nodes within an element (element mean) was used for interpreting stress results in subsequent simulations, as illustrated in Figure 19.

## 5. Simulation Results

### 5.1. Moisture Diffusion and Moisture–Thermal Stress Results

**Figure 20:**
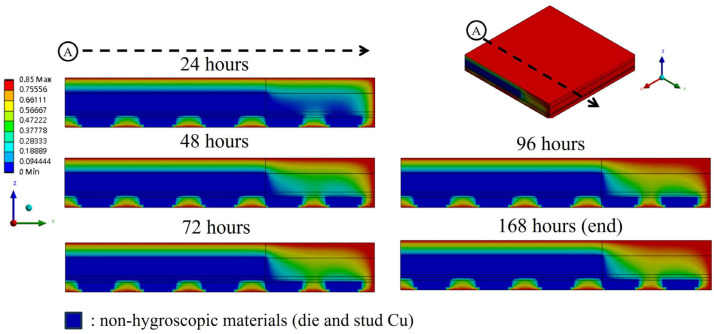
Moisture diffusion results under MSL 1 testing conditions.

Figure 20 shows moisture diffusion results at MSL 1. The blue areas represent zero moisture concentration and non-hygroscopic materials, while the red areas represent the results of complete saturation under 85% RH environmental humidity. Moisture mainly diffuses from the surface of the EMC material inward, with the entire package stabilizing after approximately 96 h. Moisture diffused more slowly in the thicker EMC because of the distance of exposure to the environment and the lower diffusivity of the material.

It is important to note that although von Mises stress is often used in general stress evaluation, the observed stress concentration consistently occurs at the die corners, a region highly prone to fracture or delamination. Therefore, the subsequent analysis focuses on maximum principal stress, which is more suitable for evaluating brittle failure modes such as die cracking. In all cases, tensile stresses are observed within the die region, reinforcing the likelihood of crack initiation or interfacial delamination at the die corners under MSL and reflow conditions.

**Figure 21:**
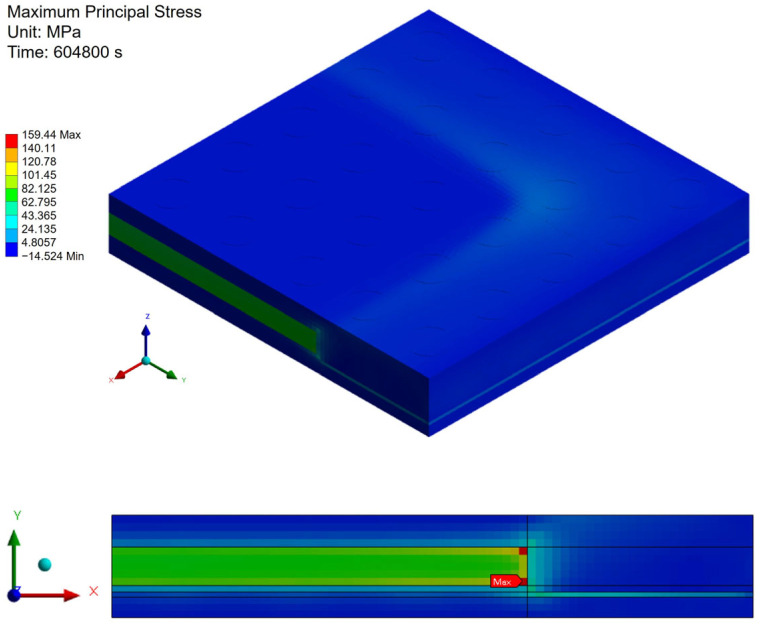
Thermal stress induced by temperature effect.

**Figure 22:**
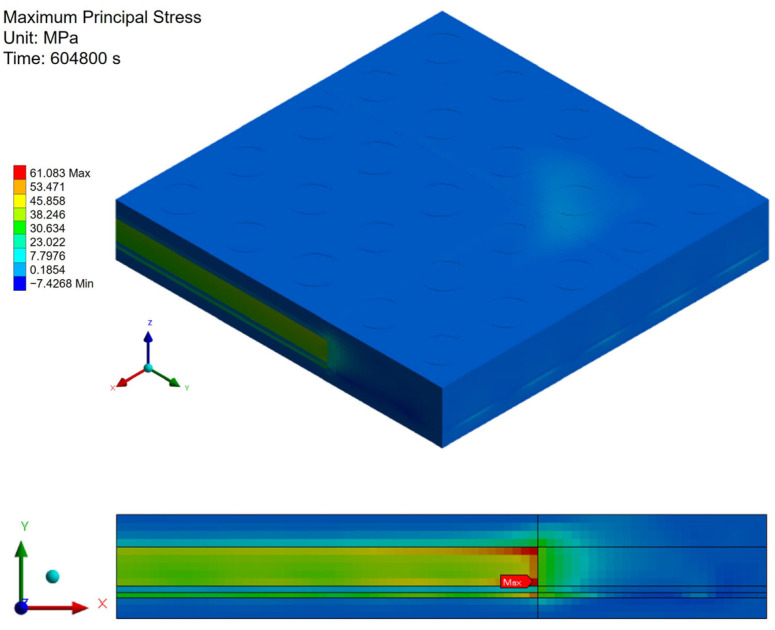
Moisture stress induced by humidity effect.

**Figure 23:**
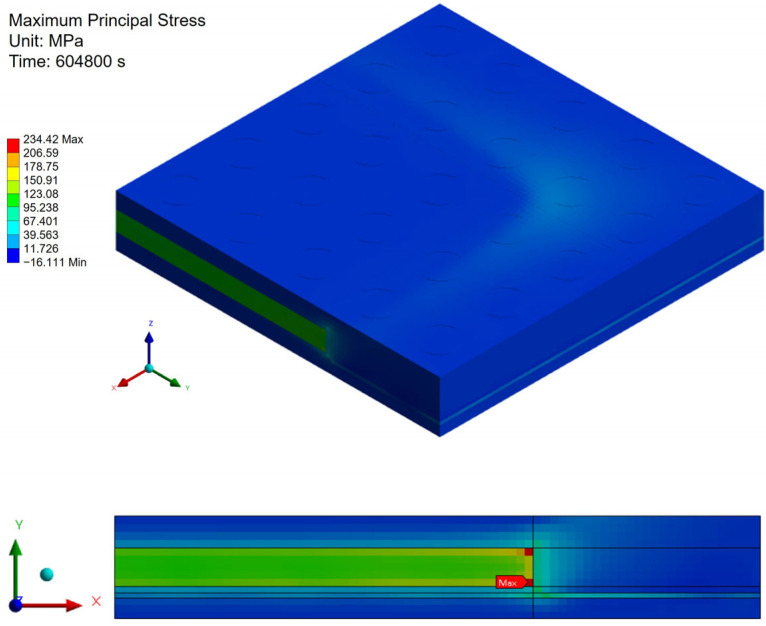
Hygrothermal stress induced by temperature and humidity effects.

Figure 21, Figure 22 and Figure 23 present the distribution of maximum principal stress at 168 h under three separate loading conditions: temperature only, humidity only, and combined hygrothermal loading. When considering only humidity, the maximum principal stress reaches 61 MPa. Under combined hygrothermal loading, the maximum principal stress increases significantly to 234.4 MPa and is located at the corner of the die. These results confirm that thermal strain contributes the most to stress generation, while moisture effects further amplify the stress distribution.

### 5.2. Moisture–Thermal–Vapor Stress Analysis During Reflow

During reflow processes, we evaluated the internal vapor pressure generated due to sudden vaporization of moisture in high-temperature conditions. The thermal, moisture, and vapor pressure strains were calculated separately as [7]

(7) $$\varepsilon_{\mathrm{th}}=\alpha_{T}\;\Delta T,\;\varepsilon_{h}=ꞵC_{\mathrm{sat}}\;\times \;w,\;\varepsilon_{p}=(\frac{1-2v}{E}P_{\mathrm{sat}})\;\times \;W$$

*ε_thv_* = (*ε_th_* + *ε_p_*) + *ε_h_* = *ε_eq_* + *ε_h_* (8)

(9) $$\alpha_{\mathrm{eq}}=\frac{\varepsilon_{\mathrm{eq}}}{\Delta T}\;=\alpha_{T}+(\frac{1-2v}{E\Delta T}P_{\mathrm{sat}})\times \;W$$

*ε_th_*: thermal strain; *ε_h_*: hygroscopic strain; *ε_p_*: vapor strain; *ε_eq_*: equivalent strain; *α_T_*: the coefficient of thermal expansion; Δ*T*: temperature difference; *ꞵ*: the coefficient of moisture expansion; *W*: relative humidity; *ε_thv_*: total strain; *α_eq_*: the equivalent coefficient of expansion; *v*: Poisson’s ratio; *E*: Young’s modulus; *P_sat_*: vapor pressure.

**Figure 24:**
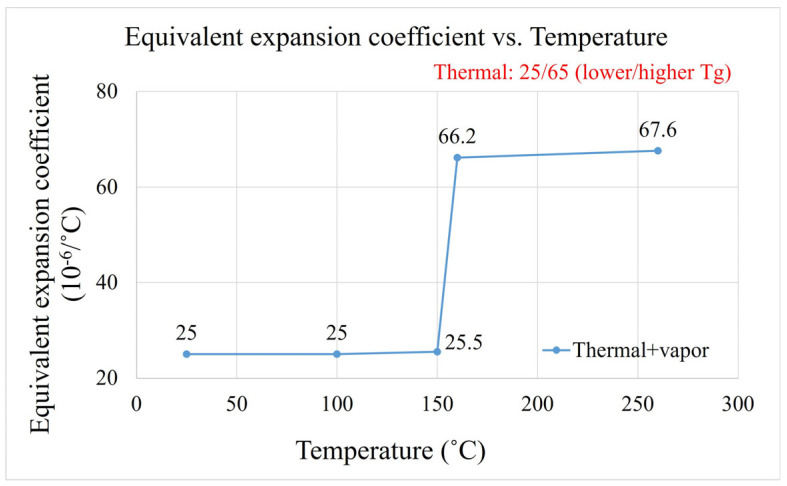
Equivalent expansion coefficient at different temperatures.

The thermal expansion coefficients above and below the EMC material’s glass transition temperature (*T_g_*) are 25 and 65 (unit: 10^−6^/°C), respectively. Figure 24 shows the calculated equivalent expansion coefficient.

Based on JEDEC specifications, the temperature reaches 260 °C during the reflow process. To ensure accuracy in the high-temperature simulation, the temperature-dependent mechanical behavior of the EMC material must be considered. Specifically, the Young’s modulus of the EMC decreases significantly when the temperature exceeds its glass transition temperature (*T_g_*). In this study, the reference values for the high-temperature modulus are those reported by Shih [20]. According to their results, the Young’s modulus of the EMC material drops from 20 GPa (below *T_g_*) to 3.4 GPa (above *T_g_*). These values were adopted in the simulation to better capture the mechanical response under reflow conditions.

**Figure 25:**
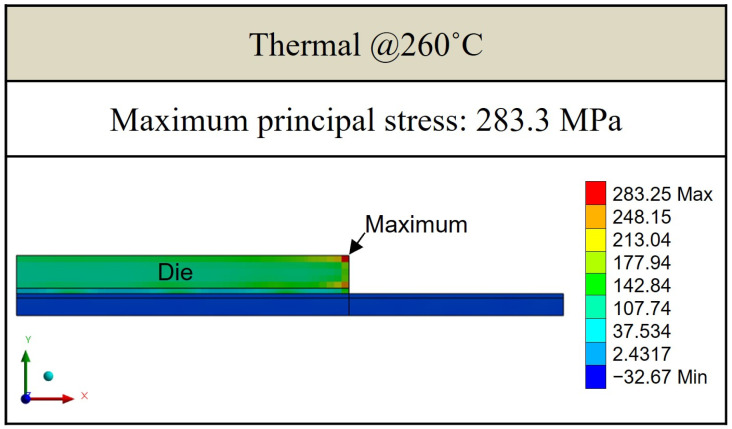
Principal stress distribution induced by thermal effect under reflow conditions.

**Figure 26:**
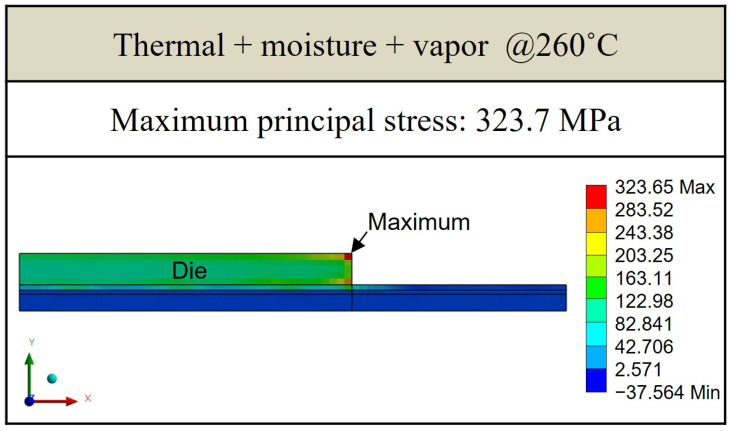
Principal stress distribution induced by thermal–moisture–vapor effects under reflow conditions.

Figure 25 and Figure 26 show the results of maximum principal stress at reflow temperatures, with and without considering humidity and vapor pressure effects. It is observed that the maximum principal stress consistently occurs at the die corners. While there are minor differences in stress magnitude when humidity and vapor are included, the dominant contribution to stress remains the thermal effect due to the large temperature gradient and resulting thermal strain.

**Figure 27:**
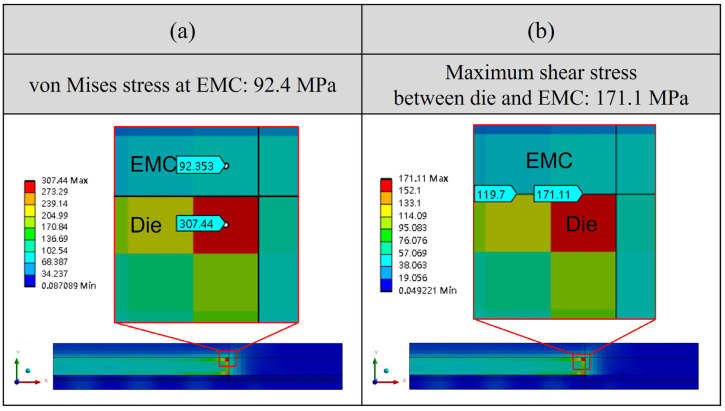
Maximum stress distribution of (**a**) von-Mises stress and (**b**) maximum shear stress between die and EMC.

Figure 27 shows the stress distribution at reflow temperatures, providing additional information regarding potential reliability issues. As the maximum stress appears at the die corner, which corresponds to the interface between the die and EMC, this region is identified as a potential location for delamination failure. According to the simulation results, the von Mises stress within the EMC material reaches 92.4 MPa, and the maximum shear stress at the die and EMC interface is 171.1 MPa. These values suggest that significant interfacial stress may develop during reflow due to thermal effects, which could compromise long-term reliability.

### 5.3. Structure Parameter Sensitivity Analysis

**Figure 28:**
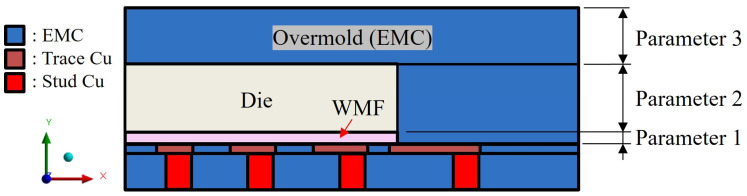
Cross-section schematic of single package.

To evaluate the impact of structural configuration on stress development in FOPLP, this study conducted a sensitivity analysis by varying the thickness of three key components—the overmold (EMC), die, and WMF—as illustrated in Figure 28. Seven geometric models with different layer thicknesses were established to investigate how these variations influence stress distribution during reflow. The specific structural parameters used in the sensitivity analysis are summarized in Table 3.

**Figure 29:**
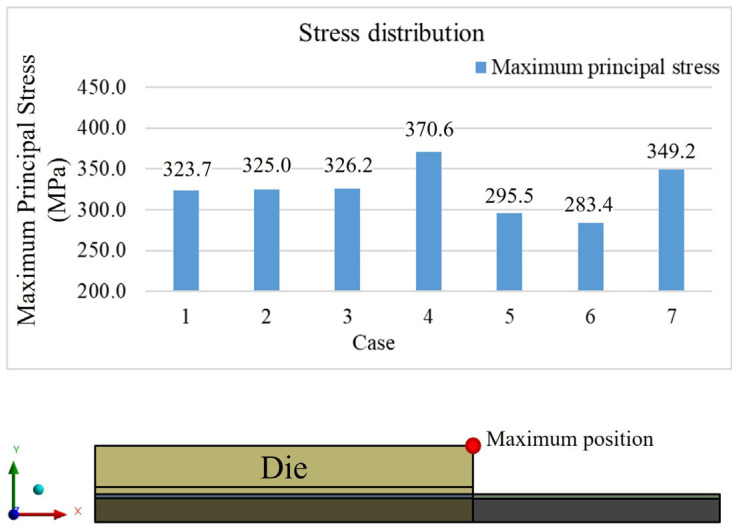
Maximum stress value results from seven cases.

**Figure 30:**
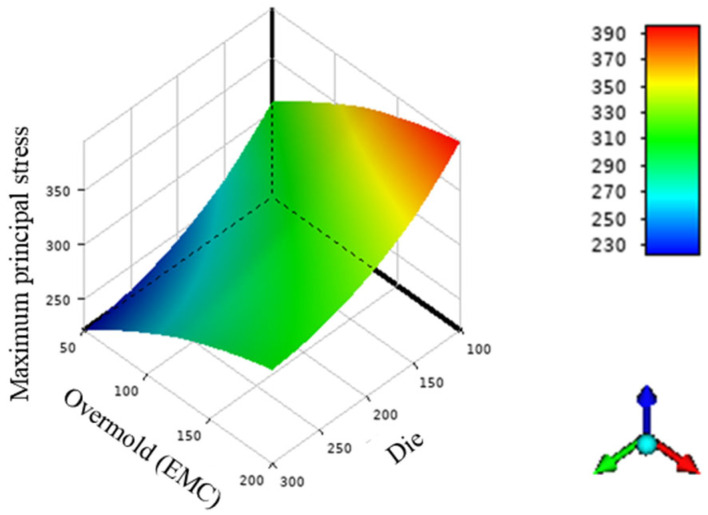
Maximum principal stress values with different die and overmold thicknesses.

According to the stress results from the seven different models, the maximum stress values are located at the die’s corner, as shown in Figure 29 and Figure 30. From cases 1, 2, and 3, it is observed that adjusting the thickness of the WMF material has a minimal influence on the maximum stress value. Comparing cases 1, 5, and 6 reveals that reducing the EMC thickness and increasing the die thickness help to lower the maximum stress value.

**Figure 31:**
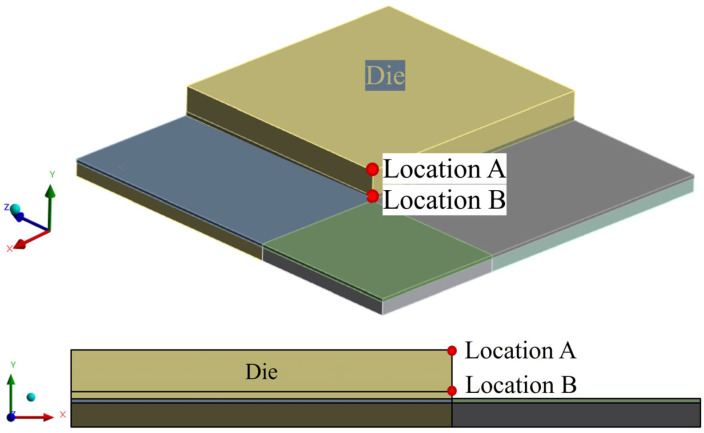
Schematic of stress value locations.

Additionally, a local sensitivity analysis was conducted using the response surface constructed from the seven individual design cases, focusing on Locations A and B as shown in Figure 31. The local sensitivity values were calculated using the Response Surface Method module in ANSYS Workbench 2023 R1. These values indicate the relative impact of each structural parameter, specifically the thicknesses of the overmold, die, and WMF, on the maximum principal stress.

**Figure 32:**
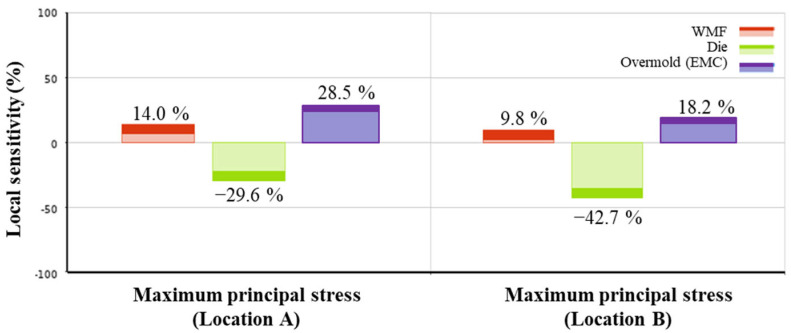
Sensitivity analysis results.

Each sensitivity value is calculated with respect to the baseline model, which corresponds to the original design configuration. A positive sensitivity means that increasing the parameter leads to higher stress, while a negative value indicates the opposite. For instance, increasing the overmold thickness at Location A results in a local sensitivity of 28.5%, whereas the die thickness shows a negative sensitivity of 29.6%, suggesting that reducing the die thickness lowers the local stress. Due to its relatively small thickness, the WMF shows lower sensitivity in both locations. These findings are consistent with those reported by Xue [21], as illustrated in Figure 32.

## 6. Conclusions

This study presents a comprehensive hygrothermal stress analysis methodology for a single-package molding-first FOPLP structure under high-temperature and high-humidity conditions. By integrating experimental measurements and finite element analysis, the effects of moisture and thermal loading on package reliability were systematically investigated. The key findings and contributions are summarized as follows:

1. Experimental characterization using thermal–mechanical analysis under various temperature and humidity conditions revealed that moisture desorption at elevated temperatures significantly reduces strain, with observable differences across EMC materials. These findings provided quantitative input for simulation parameters.
2. Under JEDEC MSL 1 conditions, moisture diffusion within the EMC was simulated, showing that the package reaches moisture saturation after approximately 96 h at 85% relative humidity. The simulation identified the die corners as critical stress concentration areas, with the maximum principal stress reaching 234.4 MPa under combined thermal and moisture loading, compared to 61.1 MPa under moisture-only conditions.
3. During the reflow process, stress values increased further due to thermal effects. Simulations showed that the maximum principal stress was 283.3 MPa under thermal-only loading and 326.7 MPa when vapor pressure was also considered, confirming temperature as the dominant factor in stress evolution.
4. Structural sensitivity analysis demonstrated that die and EMC thicknesses have a significant influence on stress levels. Increasing die thickness or reducing EMC thickness led to a reduction of up to 12.4% in maximum principal stress, offering effective design strategies for reliability enhancement.

In conclusion, this study proposes an integrated hygrothermal analysis workflow that integrates experimentally measured material properties into simulations, enabling accurate prediction of failure-prone regions and stress magnitudes in FOPLP structures. The combined approach not only improves understandings of moisture–thermal interactions but also provides practical design guidelines to mitigate stress-induced failures and improve long-term reliability. In addition, the sensitivity analysis framework employed in this study, based on the Response Surface Method within ANSYS Workbench, offers strong potential for industrial scalability. By integrating this approach with FEA workflows, the methodology can be extended to support early-stage design decisions across a wide range of package architectures.

## Figures and Tables

**Table 1:** Material properties used in moisture analysis.

Material	Die	Cu	WMF	EMC 1
Young’s modulus (GPa)	131	120	9	20
Coefficient of thermal expansion (10^−6^/°C)	2.8	17	16/23 (lower/higher *T_g_*)	25/65 (lower/higher *T_g_*)
Poisson’s ratio	0.28	0.36	0.3	0.3
Density (g/cm^3^)	2.33	8.92	0.9	1.996
Diffusivity (m^2^/s)	-	-	3.94 × 10^−12^	1.391 × 10^−13^
Saturated concentration (kg/m^3^)	-	-	12.5	5.625
Coefficient of moisture expansion (m^3^/kg)	-	-	7.32 × 10^−5^	1.7 × 10^−4^ @ 85 °C 2.5 × 10^−4^ @ 260 °C

**Table 2:** Different mesh size and number of elements.

Mesh Size (μm)	Number of Elements
60	40,356
50	58,410
40	94,163
35	149,685
30	176,545
27.5	211,680

**Table 3:** Structural parameters for sensitivity analysis.

	Parameters	Parmeters 1: WMF (μm)	Parmeters 2: Die (μm)	Parmeters 3: Overmold (μm)
Case				
1 (original)		25	150	125
2		35	150	125
3		45	150	125
4		25	100	125
5		25	200	125
6		25	150	75
7		25	150	175

## Data Availability

The original contributions presented in this study are included in the article. Further inquiries can be directed to the corresponding author.

## References

[1] Motohashi N., Kimura T., Mineo K., Yamada Y., Nishiyama T., Shibuya K., Kobayashi H., Kurita Y., Kawano M. System in Wafer-Level Package Technology with RDL-first Process. Proceedings of the IEEE 61st Electronic Components and Technology Conference.

[2] Chen S., Wang S., Hunt J., Chen W., Liang L., Kao G., Peng A. A Comparative Study of a Fan-Out Packaged Product: Chip First and Chip Last. Proceedings of the IEEE 66th Electronic Components and Technology Conference.

[3] Lau J.H., Ko C.-T., Yang K.-M., Peng C.-Y., Xia T., Lin P.B., Chen J., Huang P.P.-C., Liu H.-N., Tseng T.-J. (2020). Panel-Level Fan-Out RDL-first Packaging for Heterogeneous Integration. IEEE Trans. Compon. Packag. Manuf. Technol..

[4] Wong E.-H., Park S. (2016). Moisture Diffusion Modeling—A Critical Review. Microelectron. Reliab..

[5] Wong E.-H., Teo Y.-C., Lim T.-B. Moisture Diffusion and Vapor Pressure Modeling of IC Packaging. Proceedings of the IEEE 48th Electronic Components and Technology Conference.

[6] Chen W.-C. (2001). The Study of Precondition Variations on the Warpage of PBGA Packages. Master’s Thesis.

[7] Tee T.-Y., Zhong Z. (2004). Integrated Vapor Pressure, Hygroswelling, and Thermo-Mechanical Stress Modeling of QFN Package During Reflow with Interfacial Fracture Mechanics Analysis. Microelectron. Reliab..

[8] Lwo B.-J., Lin C.-S. (2007). Measurement of Moisture-Induced Packaging Stress with Piezoresistive Sensors. IEEE Trans. Adv. Packag..

[9] Tsai M.-H., Hsu F.-J., Weng M.-C., Hsu H.-C. Advanced Moisture Diffusion Model and Hygro-Thermo-Mechanical Design for Flip Chip BGA Package. Proceedings of the International Conference on Electronic Packaging Technology & High Density Packaging.

[10] Wu J.-Y., Chen T.-W., Chiu T.-C., Chen D.-L., Chen T.-Y., Shih M.-K. Coupled Hygro-Thermo-Mechanical Analysis of Moisture-Induced Interfacial Stresses in Fan-Out Package. Proceedings of the 14th International Microsystems, Packaging, Assembly and Circuits Technology Conference (IMPACT).

[11] Chiu T.-C., Wu J.-Y., Liu W.-T., Liu C.-W., Chen D.-L., Shih M., Tarng D. A Mechanics Model for the Moisture-Induced Delamination in Fan-Out Wafer-Level Package. Proceedings of the IEEE 70th Electronic Components and Technology Conference.

[12] Chen Z., Feng Z., Ruan M., Xu G., Liu L. (2022). Effects of Moisture Diffusion on a System-in-Package Module by Moisture–Thermal–Mechanical-Coupled Finite Element Modeling. Micromachines.

[13] Jiang J., Ren Y.-F., Gao C.-S., Zhang G., Ye H., Fan J., Song G.-Q. Research on PCT reliability of power devices based on FOPLP. Proceedings of the 2022 23rd International Conference on Electronic Packaging Technology (ICEPT).

[14] Liang C.-W., Sung Y.-C., Hwang S.-J., Shih M.-H., Liao W.-H., Lin T.-H., Yang D.-Y. (2024). Fan-out panel-level package warpage and reliability analyses considering the fabrication process. J. Manuf. Process..

[15] JEDEC Solid State Technology Association (2021). J-STD-020: Moisture/Reflow Sensitivity Classification for Nonhermetic Surface Mount Devices.

[16] Hsu F.-J. (2010). Thermal-Hygro-Vapor Pressure-Mechanical Analysis and Reliability on MEMS-Based Pressure Sensor. Master’s Thesis.

[17] Wong E.-H., Rajoo R., Koh S., Lim T.-B. (2002). The Mechanics and Impact of Hygroscopic Swelling of Polymeric Materials in Electronic Packaging. J. Electron. Package.

[18] Hong Y.-T. (2018). Thermo-Hygro-Mechanical Stress and Warpage Analyses of Fan-Out Wafer Level Package. Master’s Thesis.

[19] Gao R., Ma R., Li J., Wang Q., Cao L., Su M. Characterization and Analysis of Moisture Absorption in Embedded System in Packaging. Proceedings of the IEEE 72nd Electronic Components and Technology Conference.

[20] Shih M.-K., Huang C.-Y., Chen T.-H., Wang C.-C., Tarng D., Hung C.-P. (2019). Electrical, thermal, and mechanical characterization of eWLB, fully molded fan-out package, and fan-out chip last package. IEEE Trans. Compon. Packag. Manuf. Technol..

[21] Xue K., Wu J.-S., Chen H.-B., Gai J.-B., Lam A. Reliability Based Design Optimization for Fine Pitch Ball Grid Array: Modeling Construction and DOE Analysis. Proceedings of the 11th Electronics Packaging Technology Conference.

